# Variants in Maternal Effect Genes and Relaxed Imprinting Control in a Special Placental Mesenchymal Dysplasia Case with Mild Trophoblast Hyperplasia

**DOI:** 10.3390/biomedicines9050544

**Published:** 2021-05-13

**Authors:** Tien-Chi Huang, Kung-Chao Chang, Jen-Yun Chang, Yi-Shan Tsai, Yao-Jong Yang, Wei-Chun Chang, Chu-Fan Mo, Pei-Hsiu Yu, Chun-Ting Chiang, Shau-Ping Lin, Pao-Lin Kuo

**Affiliations:** 1 Institute of Biotechnology, National Taiwan University, Taipei 106, Taiwan; tienchi.huang@gmail.com (T.-C.H.); win850310@gmail.com (J.-Y.C.); b87606029@ntu.edu.tw (W.-C.C.); josefinemok@gmail.com (C.-F.M.); 2 Department of Pathology, National Cheng Kung University Hospital, Tainan 704, Taiwan; changkc@mail.ncku.edu.tw; 3 Department of Radiology, National Cheng Kung University Hospital, Tainan 704, Taiwan; n506356@mail.hosp.ncku.edu.tw; 4 Department of Pediatrics, National Cheng Kung University Hospital, Tainan 704, Taiwan; yaojong@mail.ncku.edu.tw; 5 Department of Obstetrics and Gynecology, National Cheng Kung University Hospital, College of Medicine, National Cheng Kung University, Tainan 704, Taiwan; pumkinfat56@gmail.com; 6 Department of Obstetrics and Gynecology, Kuo General Hospital, Tainan 700, Taiwan; 7 Department and Graduated Institute of Forensic Medicine, College of Medicine, National Taiwan University, Taipei 106, Taiwan; altanasia.chiang@gmail.com; 8 Agricultural Biotechnology Research Center, Academia Sinica, Taipei 115, Taiwan; 9 Research Center for Developmental Biology and Regenerative Medicine, National Taiwan University, Taipei 106, Taiwan; 10 Center for Systems Biology, National Taiwan University, Taipei 106, Taiwan

**Keywords:** placental mesenchymal dysplasia, hydatidiform mole, genomic imprinting, maternal effect genes, trophoblast

## Abstract

Placental mesenchymal dysplasia (PMD) and partial hydatidiform mole (PHM) placentas share similar characteristics, such as placental overgrowth and grape-like placental tissues. Distinguishing PMD from PHM is critical because the former can result in normal birth, while the latter diagnosis will lead to artificial abortion. Aneuploidy and altered dosage of imprinted gene expression are implicated in the pathogenesis of PHM and also some of the PMD cases. Diandric triploidy is the main cause of PHM, whereas mosaic diploid androgenetic cells in the placental tissue have been associated with the formation of PMD. Here, we report a very special PMD case also presenting with trophoblast hyperplasia phenotype, which is a hallmark of PHM. This PMD placenta has a normal biparental diploid karyotype and is functionally sufficient to support normal fetal growth. We took advantage of this unique case to further dissected the potential common etiology between these two diseases. We show that the differentially methylated region (DMR) at NESP55, a secondary DMR residing in the *GNAS* locus, is significantly hypermethylated in the PMD placenta. Furthermore, we found heterozygous mutations in *NLRP2* and homozygous variants in *NLRP7* in the mother’s genome. *NLRP2* and *NLRP7* are known maternal effect genes, and their mutation in pregnant females affects fetal development. The variants/mutations in both genes have been associated with imprinting defects in mole formation and potentially contributed to the mild abnormal imprinting observed in this case. Finally, we identified heterozygous mutations in the X-linked *ATRX* gene, a known maternal–zygotic imprinting regulator in the patient. Overall, our study demonstrates that PMD and PHM may share overlapping etiologies with the defective/relaxed dosage control of imprinted genes, representing two extreme ends of a spectrum.

## 1. Introduction

Placental mesenchymal dysplasia (PMD) is a rare clinical condition often misdiagnosed as partial hydatidiform mole (PHM) [1,2,3,4,5]. Both PMD and PHM are characterized by placentomegaly and grapelike cystic structures intermingled with morphologically normal placental tissues on gross appearance [6]. This resemblance has led PMD to be described as a pseudopartial mole [3]. However, in contrast to molar pregnancies, which rarely result in viable infants, pregnancies with PMD usually result in normal deliveries, but some cases show complications such as intrauterine growth restriction, fetal anemia, or even neonatal death [7]. Since there is a significant percentage of viable fetuses with PMD, it is important to distinguish PMD and PHM pregnancies to avoid unnecessary abortions and to evaluate the potential risk of persistent gestational trophoblast disease in patients.

Morphologically, the major difference between PMD and PHM is that PMD affects only the mesenchymal lineage of the placenta and lacks the typical trophoblast hyperplasia shown in molar pregnancies [6]. Normal PMD could potentially be differentiated from PHM by diagnosing the difference in the disease location with magnetic resonance imaging [8,9]. At the genetic level, molar pregnancies are associated with imprinting defects, which result in unbalanced dosages of parental-origin-specific expressed imprinted genes and their associated networks [10]. For example, most complete hydatidiform moles (CHMs) are androgenetic and have diploid genomes containing two copies of the paternal genomes. Conversely, PHM usually results from a triploid zygote with two copies of the paternal genome and one copy of the maternal genome [10]. Unlike PHM, the karyotype for most PMDs is diploid (most commonly 46 XX), although aneuploidy has also been reported [11]. It is not clear to what extent deregulated imprinted gene expression is associated with the etiology of PMD. Additionally, Kaiser-Rogers et al. proposed androgenetic/biparental mosaicism as a possible cause for PMD formation [12]. Furthermore, up to one-third of PMD cases are associated with Beckwith Wiedemann syndrome (BWS), which is identified as an imprinting disorder [13]. These findings imply that the deregulation of certain imprinted gene expression may be part of the etiology of the PMD phenotype.

Several critical imprinting regulators fall into the categories of “maternal effect genes” or “maternal–zygotic genes”. Maternal effect genes are determined by whether the mother carries a mutant gene that influences the development of oocytes and embryos. The maternal effect is a situation in which the genotype of the mother and the environment can determine the phenotype of the offspring, regardless of the offspring’s genotype [14,15]. *NLRP2* and *NLRP7* mutations in the mother cause imprinting defects in the offspring, including BWS or reproductive failures [16,17,18]. *ZFP57* and *ZFP445* are two examples of maternal–zygotic genes translated into the ZFP57/ZFP544 protein complex necessary for the maintenance of genomic imprinting marks on imprinting control regions [19].

Here, we report a rare PMD case diagnosed by dilated blood vessels with fibrin deposits and myxedematous with stromal fibrosis in enlarged villi. The positive staining for desmin and decreased actin expression in placental stromal cells of enlarged (i.e., dysplastic stem) villi were consistent with the diagnosis of PMD. The defective placenta also demonstrated focal trophoblastic hyperplasia, which is usually associated with molar pregnancies. Genetic analysis showed that both placental tissues and the living fetus contained a biparental diploid (46, XX) karyotype without detectable androgenetic mosaicism. Notably, this pregnancy leads to a grossly healthy child except for liver hamartoma, which is also a characteristic complication found in PMD-associated babies [20].

Since imprinted genes are important for proper placentation, imprinting defects have implications in many placental diseases [21,22,23]. We would like to use this reported PMD case with PHM-associated characteristics to test the hypothesis that PMD and molar pregnancy may represent a spectrum of imprinting deregulation that can result in overlapping phenotypes with different levels of severity. We isolated DNA samples from cystic and adjacent normal-appearing placental tissue along with fetal cord blood and analyzed the presence/absence of differentially methylated patterns of the imprinting control regions (ICRs) at several imprinted loci, including those associated with BWS. We compared the differentially methylated regions (DMRs) between patient tissue and control healthy tissue. In addition, we analyzed the patient’s genetic sequences for maternal effect genes that are known to have implications in controlling genomic imprinting.

By combining pathology, imprinting and genetic mutation, we attempt to clarify the cause of this reported case and to enhance the accuracy of clinical diagnosis. Whether PMD and molar pregnancies represent a spectrum of various placental mesenchymal/trophoblast diseases will be discussed.

## 2. Materials and Methods

### 2.1. Clinical History

**Figure 1:**
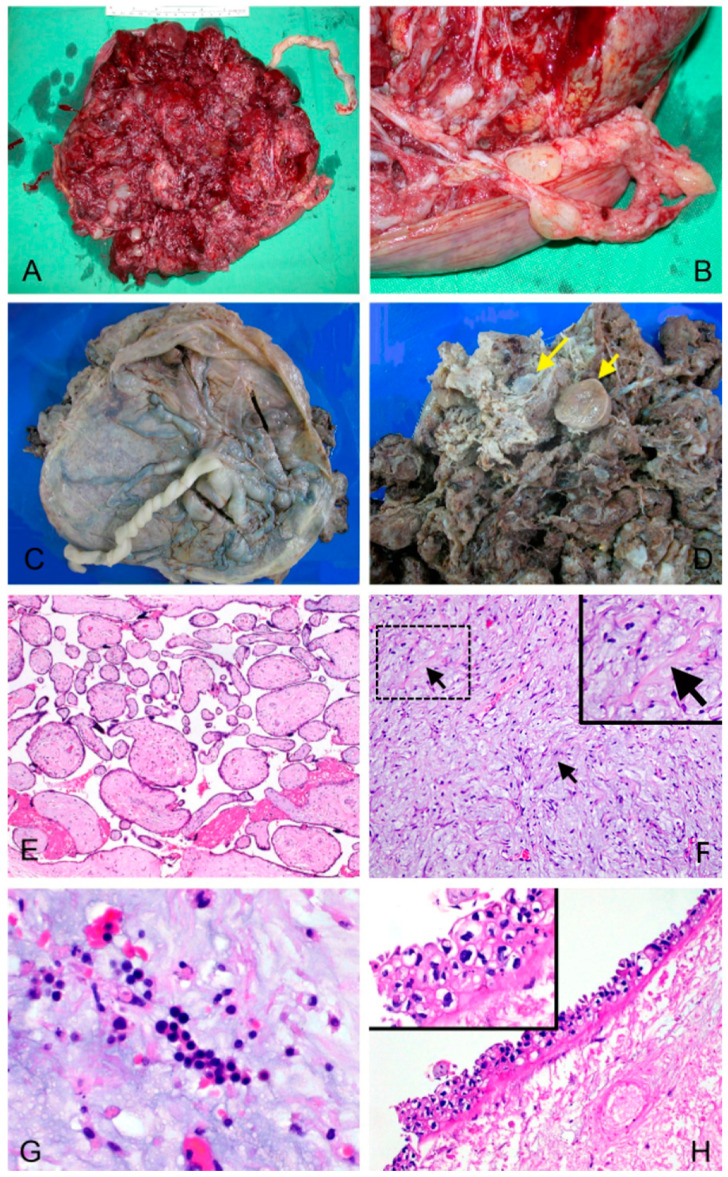
Gross appearance and histopathology of the patient’s placenta. (gross, (**A**–**D**); microscopic (**E**–**H**)) (**A**,**B**) The placenta measured 25 × 20 × 3.5 cm and weighed 1350 g. An umbilical cord measuring 31 cm long was inserted centrally at the fetal side of the placenta. (**C**) The fetal surface of the membrane showed normal translucency without meconium staining. (**D**) The maternal surface revealed minor calcification, intact cotyledons, and no evidence of retroplacental hemorrhage. Focal infarction, hydropic changes in villi (long arrow) and enlarged villi with consolidation (2–5 cm in diameter, short arrow) were noted. The umbilical cord contained two arteries and one vein, and presented no evidence of knots, adhesions, or thromboses. Focal torsion was noted. (**E**–**H**) Under magnification, the placenta without gross edema or infarction showed normal histology (**E**). The membrane showed a normal chorion and amnion without evidence of infection. Conversely, the grossly enlarged villi showed edematous changes with stromal fibrosis (bold arrows; the pinkish area) (**F**), fetal nucleated erythrocytes (**G**), focal trophoblastic hyperplasia with atypia ((**H**) and inset), central cisterns, and a scalloped villous outline. (**E**) H&E, 40×; (**F**) H&E, 200×; (**G**) H&E, 400×; (**H**) H&E, 200×, inset, 400×.

A 30-year-old Taiwanese woman, Gravida, experienced two consecutive early spontaneous abortions (<12 weeks). Of the two previous miscarried fetuses, one baby was male, and the sex of the other baby was unknown. However, these conceptuses from prior pregnancies were not characterized further. She was referred to our special clinic at 15 weeks of gestation in her third pregnancy due to abnormal maternal screening results of potential fetal aneuploidy. Her serum fetoprotein level was 4.57 times the median (MoM), and her beta-HCG level was 12 MoM. The estimated Down syndrome risk was 1 in 126, and the risk for neural tube defects was 1 in 14. Ultrasound examination revealed a singleton pregnancy with no fetal structural abnormalities and a large placenta with numerous echo-lucent vesicles, indicating a hydatidiform mole (HM) with a co-existing fetus. Amniocentesis with concomitant chorionic villus sampling (CVS) was performed for karyotyping of the fetus and placental tissue. The karyotype of the amniocytes showed a normal female karyotype (46, XX). Serial ultrasound examinations and beta-HCG level determinations were performed at 3-week intervals. Chest X-ray examinations and thyroid function tests were performed at monthly intervals. The patient’s beta-HCG levels were constantly high throughout the gestational period, ranging from 60,000 to 160,000 mIU/mL. Ultrasound examination showed progressive enlargement of the placenta and normal fetal growth. A healthy normal female infant was delivered at the gestational age of 36 weeks. The baby weighed 2625 g with an Apgar score of 9 at the first minute and 10 at the fifth minute. The placenta weighed 1350 g (Figure 1A). The maternal serum beta-HCG level was regularly traced and became undetectable 4 months after delivery. The patient was free of disease for two years and became pregnant again. The gestational course was uneventful this time, and she delivered a healthy female baby at the gestational age of 37 weeks.

### 2.2. STR Analysis

Informed consent for genetic investigations was obtained from the couple. The chromosomal constitutions of the umbilical cord and the gross vesicle of cystic tissue were analyzed using G-banding by the Trypsin–Giemsa (GTG) method. DNA samples were extracted from peripheral blood lymphocytes from the parents, cystic tissue from the placenta, and umbilical cord blood representing the genotype of the baby, and were analyzed using short tandem repeat (STR) markers on the AmpF/STR—Plus^TM^ zygosity determination system (Applied Biosystems, Foster City, CA, USA).

### 2.3. Analysis of Methylation Status in Parental-Origin-Specific Differentially Methylated Regions

DNA samples used for methylation analysis included: the cystic region of the PMD placenta; the morphologically normal tissue of this placenta; fetal cord blood and DNA sample for the “unrelated normal control placentae” mixed with equal portions of DNA samples from 3 other unrelated normal pregnancies all with female babies and of the same gestational week. Bisulfite conversion treatment of fetal cord DNA was performed with the EZ DNA Methylation-Gold Kit (Zymo Research, Irvine, CA, USA). Briefly, 0.5–1.0 μg of genomic DNA was treated with 9 M sodium bisulfite, denatured at 98 °C for 10 min, and converted at 64 °C for 2.5 h. Samples were collected by a microcolumn and desulfonated with 0.3 M NaOH. After desulfonation, DNA was eluted in 10–20 μL of water. Eluted samples were amplified by PCR for various differentially methylated regions (DMRs) associated with differentially imprinted loci. The primer sequences are summarized in Supplementary Table S1. The amplified PCR products were cloned into the pGEMT Easy Vector (Promega, Madison, WI, USA) and subjected to sequence analysis.

### 2.4. Mutation Analysis of the NLRP7, NLRP2, and ATRX Genes by Direct Sequencing

Genomic DNA was extracted from the peripheral blood of the patient using the DNeasy Blood and Tissue Kit (Qiagen, Venlo, The Netherlands). The PCR conditions and primer sequences used to amplify each amplicon of NLRP7 are summarized in [24]. Purified PCR products were subjected to direct sequencing in forward and reverse directions. NLRP7 sequences from the patient were compared to those in GenBank NC_000019. All exons of NLRP2 were also amplified from the DNA samples of the patient and were sequenced to detect potential mutations. Primer sequences for NLRP2 exon sequencing are listed in Supplementary Table S2. Sanger sequencing was also performed for the exons of various other maternal effect genes and imprinting modulators, including ATRX and ZNF445/ZFP445, and sequences were compared with the gnomAD database regarding the predicted function of each mutation/variant and the frequencies in different human populations.

### 2.5. Histological and Immunohistochemical Stains

All specimens were fixed in 10% neutral formalin and embedded in paraffin. Hematoxylin and eosin (H&E) staining was performed using standard methods. Immunohistochemical (IHC) analysis was performed using a standard avidin-biotin-peroxidase complex technique and formalin-fixed, paraffin-embedded tissue sections. Microwave-enhanced antigen retrieval using 10 mM sodium citrate buffer (pH 6.0) was performed. IHC analysis was performed using an automated IHC stainer (BenchMark XT, Ventana Medical Systems, Inc., Tucson, AZ, USA). The primary antibodies and working dilutions were as follows: desmin (1:250, D33, mouse monoclonal, Dako, Glostrup, Denmark), smooth muscle actin (1:400, 1A4, mouse monoclonal, Dako, Glostrup, Denmark), p57 (1:200, 57P06 [KP10], mouse monoclonal, Thermo Fisher Scientific, Waltham, MA, USA), and beta-HCG (1:1500, rabbit polyclonal, Dako, Glostrup, Denmark). Appropriate positive and negative controls were used. Counterstaining was carried out with hematoxylin, and images were photographed using a digital microscope camera (DP12; Olympus Co., Tokyo, Japan) and processed in Adobe Photoshop version 7.0 software (Adobe Systems Incorporated, San Jose, CA, USA).

## 3. Results

### 3.1. A Diploid Placenta Classified as PMD with PHM-Like Morphology

**Figure 2:**
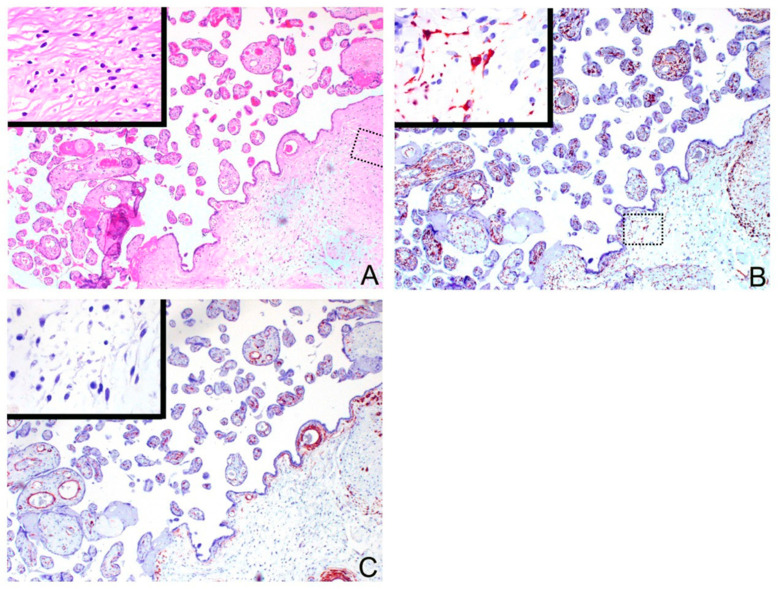
Pathological findings of placental mesenchymal dysplasia ((**A**–**C**), 40×; inset, 400×). (**A**) Hematoxylin and eosin (H&E) staining of the tissue sections. The stem villi (right lower) are enlarged with an overgrowth of fibroblasts and stromal fibrosis (inset). (**B**,**C**) Immunohistochemically, the stromal cells in the large stem villi (right lower) were positive for desmin ((**B**), inset) but negative for smooth muscle actin ((**C**), inset).

The patient’s placenta had normal tissue interspersed with diffused vesicles (Figure 1A–D). Histological examination of the placenta revealed areas of morphologically normal third-trimester chorionic villi admixed with grape-like tissue that showed mild to moderate trophoblastic hyperplasia with atypia and villous hydrops with central cistern formation (Figure 1E–H). To exclude the possibility of a complete hydatidiform mole or androgenic mosaicism, we conducted immunohistochemical staining on p57KIP2, the protein product of the maternally expressed imprinted gene cyclin-dependent kinase inhibitor 1C (*CDKN1C*), which is not expressed in cells of pure androgenetic origin. Both hydropic villi and enlarged villi (Supplementary Figure S1) were positive for p57KIP2 staining in the nuclei, excluding the contribution of pure androgenetic tissue. Moreover, immunohistochemistry showed desmin positivity but decreased actin expression in placental stromal cells of enlarged (dysplastic stem) villi, which was suggestive of PMD (Figure 2). In addition, the cells of the hyperplastic trophoblasts were mainly cytotrophoblasts with a small number of syncytiotrophoblasts, both of which were positive for beta-HCG staining (Supplementary Figure S2). Since this case lacked pathological characteristics typical of HM but showed major features of PMD, we concluded that this case is a rare entity of PMD with mild focal trophoblastic hyperplasia. This mesenchymal dysplasia-containing placenta functionally supported a fully grown fetus. This child is generally healthy except for a hepatic hamartoma (Supplementary Figure S3), which is also a characteristic complication commonly found in PMD-associated babies [20].

To elucidate whether aneuploidy or mosaicism contributed to this rare clinical case, we used the GTG method to check the karyotype of the PMD placenta and the associated baby. A normal female karyotype (46, XX) was found as the chromosomal constitution of the umbilical cord, the gross vesicles of the cystic tissue, and the adjacent normal placenta, excluding the contribution of triploid cells in any of these examined tissues. Furthermore, we used short tandem repeat (STR) markers to analyze DNA samples extracted from peripheral blood lymphocytes from the parents (paternal and maternal DNA), umbilical cord (fetus), and cystic placental tissue. Fifteen STR markers were identical between the fetus and the cystic placental tissue (Table 1). We therefore suggested that all parts of the conceptus developed from a single fertilized egg and excluded the possibility of the cystic placental tissue being mosaically triploid or aneuploid. In addition, the biparental nature of the PMD tissue was confirmed in all 12 informative loci, excluding the possibility of androgenetic or mosaicism origin of the PMD placenta.

### 3.2. Relaxation of Differential Methylation in Certain Imprinted Loci

The deregulation of imprinted genes is implicated in several types of placental disease [23]. To examine whether the imprinting marks were well established and maintained in the PMD placenta and the associated baby and to identify possible mechanisms responsible for the disease phenotypes, we used bisulfite sequencing to analyze DNA methylation at DMRs of several imprinted loci. DNA was extracted from the umbilical cord blood of the living baby, the cystic tissue, and the adjacent normal tissue in the placenta, as well as an unrelated normal control placenta.

**Figure 3:**
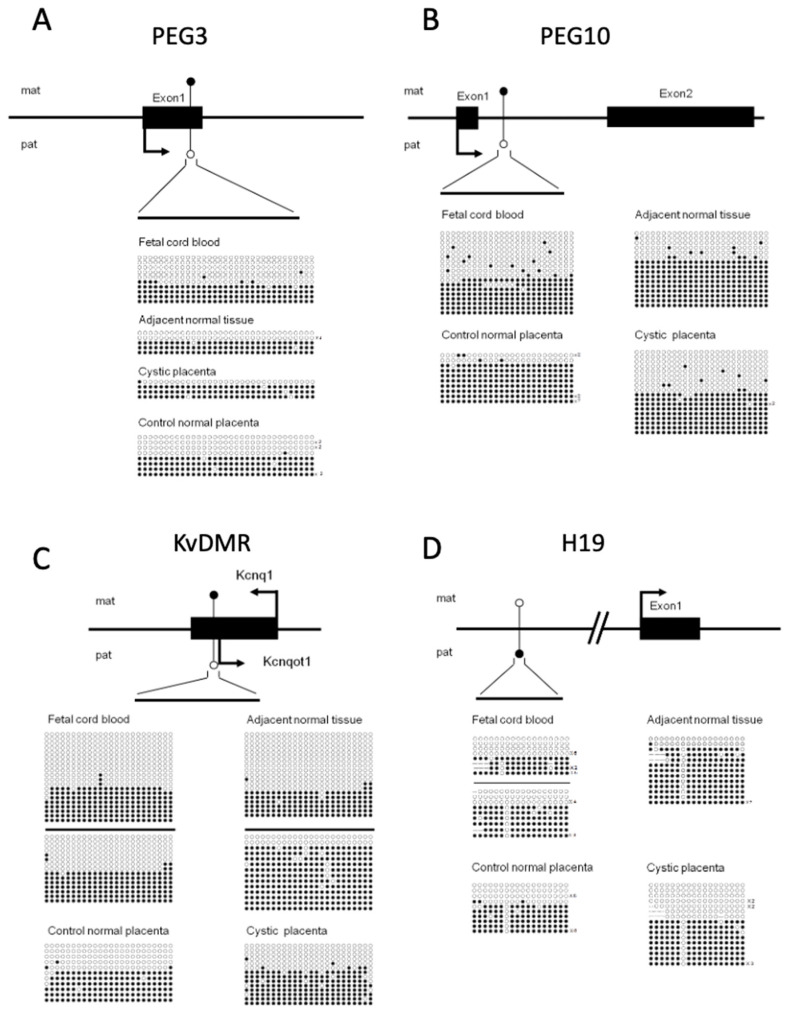
Methylation status of DMR in imprinted genes. DNA methylation profiles of maternally differentially methylated regions (DMRs), such as PEG3 (**A**), PEG10 (**B**), and KvDMR (**C**), and paternally methylated DMR H19 (**D**), were assessed by bisulfite sequencing. DNA was extracted from fetal cord blood, adjacent normal tissue, and cystic tissue in the patient’s placenta. Normal control placenta was assessed in parallel. Each line indicates a unique clone, and each circle denotes a CpG dinucleotide; filled and open circles represent methylated and unmethylated cytosines, respectively. Clones obtained from independent bisulfite treatments are separated by black lines. Solid boxes represent the coding regions. Arrows represent the directions and dominant transcripts in the indicated parental alleles. Mat: maternal allele. Pat: paternal allele.

Several maternally methylated DMRs, including PEG3-DMR, KvDMR1, and PEG10-DMR, gain CpG methylation on the maternal allele during oogenesis and remain unmethylated on the paternal allele throughout life [25]. All of these DMRs were differentially methylated in cystic PMD placental tissue, adjacent morphologically normal placental tissue, and fetal cord blood samples in our case and in independent placental tissue (Figure 3A–C). The paternally methylated H19-DMR, which is established during spermatogenesis [26], also maintained appropriate differential methylation status in all of the samples related to our special PMD case (Figure 3D). These data demonstrate appropriate establishment of imprinted marks and faithful maintenance of the methylation marks of these loci after fertilization in the embryo proper and in defective placental tissue.

**Figure 4:**
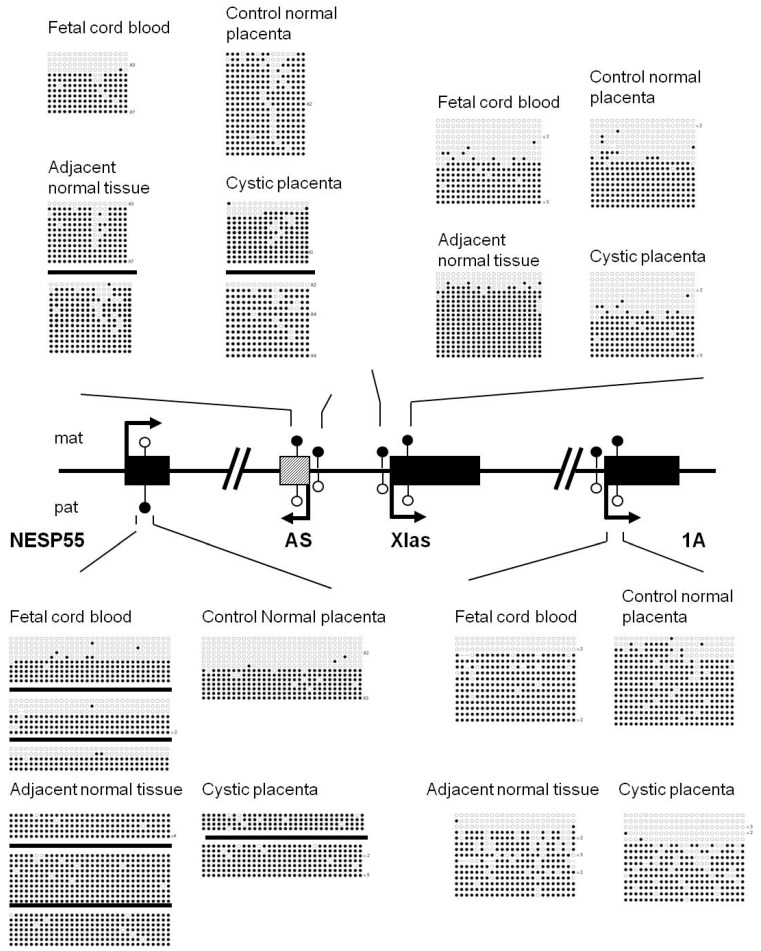
NESP55 hypermethylation in the GNAS locus. Four DMRs at the complex GNAS imprinted locus were analyzed. All DMRs are maternally methylated except for NESP55 DMR, which is a paternally methylated DMR under normal conditions. Both the cystic and morphologically normal parts of the placenta from the patient were hypermethylated in the NESP55 DMR. Mat: maternal allele. Pat: paternal allele.

We further examined four DMRs in the *GNAS* locus (Figure 4). GNAS exon 1A, GNAS antisense (AS), and XLas DMRs are maternally methylated DMRs that gain methylation during oogenesis, whereas NESP55-DMR, a paternally methylated DMR, is established after fertilization [27]. The most striking abnormality observed was the nearly fully methylated pattern on NESP55-DMR in the placental tissue of our special case, including the cystic and adjacent normal tissue. This pattern is in contrast to the differentially methylated pattern observed in the fetal cord blood of the associated baby and the independent control placenta. The strong paternalized epigenotype on the NESP55 region may be associated with the PMD and/or a mild trophoblast hyperplasia phenotype.

**Figure 5:**
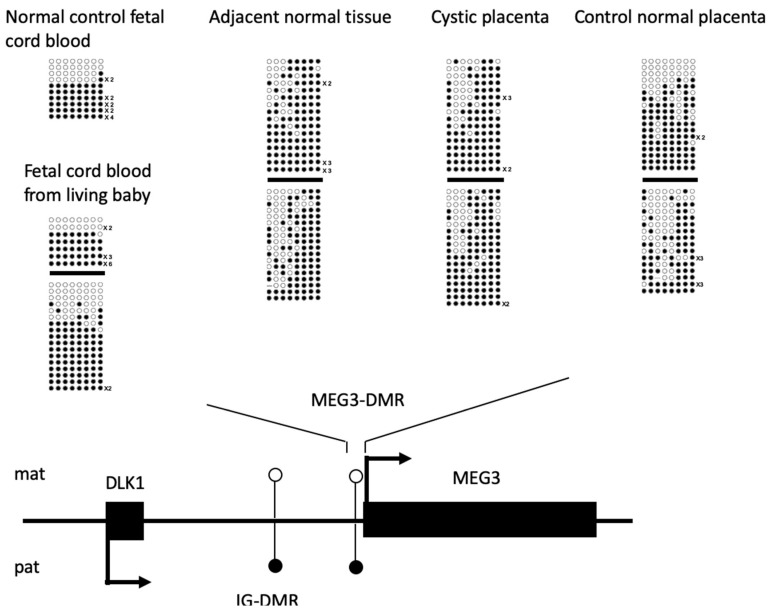
MEG3-DMR is hypermethylated in the *DLK1-DIO3* imprinted locus. The DNA methylation patterns of adjacent normal tissue and cystic placenta were different from those of normal control placenta, normal control fetal cord blood, and fetal cord blood from living babies at MEG3-DMR. Fully unmethylated clones representing epigenetically normal maternal alleles, along with relatively less methylated status, were observed in normal control placenta. There were no fully unmethylated clones from adjacent normal tissue or the cystic placenta from this pregnancy. Each line indicates a unique clone, and each circle denotes a CpG dinucleotide; filled and open circles represent methylated and unmethylated cytosines, respectively. Solid boxes represent the coding regions. Arrows represent the directions and dominant transcripts in the indicated parental alleles. Mat: maternal allele. Pat: paternal allele.

Another secondary DMR established post fertilization that we tested was the MEG3-DMR at the *DLK1-DIO3* imprinted locus. While this region had distinct differential DNA methylation patterns in fetal cord blood samples from this reported case and the independent control, the same region demonstrated mosaic DNA methylation patterns in the patient placental samples tested (Figure 5). None of the MEG3-DMR clones analyzed from the PMD-associated placenta samples were fully unmethylated. In contrast, fully unmethylated clones that could be identified from an independent control placenta sample potentially represent the normal maternal allele linked with the expression of maternally expressed MEG3 and associated downstream non-coding RNAs.

### 3.3. Mutations/Variants of Imprinting Modulating Maternal Effect Genes

Previous studies identified *NLRP7* as a maternal effect gene causing recurrent hydatidiform mole and various forms of reproductive wastage when mutated [28]. We sequenced the coding exons and the flanking introns of *NLRP7* in the patient’s genomic DNA. Compared with the reference sequence (NC_000019.8), we identified several variants and one amino acid change in exon 4 of *NLRP7* (Supplementary Figure S4). We found the missense mutation c.955G>A in exon 4, which caused an amino acid change from valine to isoleucine (V319I). We also identified four synonymous mutations, c.390G>A and c.1137G>A in exon 4, and c.2682T>C and c.2775A>G in exon 9, which did not cause amino acid changes. In addition, there were 11 other intronic DNA variants in our patient.

We further analyzed another maternal effect gene, *NLRP2*, whose mutation was found in some BWS cases [17] and which plays an important role in embryonic development. Mutations in NLRP2 result in an imprinting disorder in the offspring. The patient bears a heterozygous mutation in *NLRP2*, 19: 54983244-A/G (Thr516Ala). Based on the gnomAD database, this mutation/variant exists in 0.5% of African, 0.5% of Latin American, 0.8% of European (non-Finnish), 0.02% of East Asian, and 0.1% of South Asian populations. This particular mutation was not found in the 1517 healthy individuals in the Taiwanese population with sequences in the Taiwan Biobank Database.

We also sequenced other potential imprinting modulators, including ZNF445/ZFP445 [19], a partner of a well-known imprinting control factor, ZFP57. Similarly to ZFP57, ZNF445 also binds to many parental-specific differentially methylated regions to maintain imprinting-associated epigenetic marks, but we did not find mutations in *ZNF445* in our patient.

**Figure 6:**
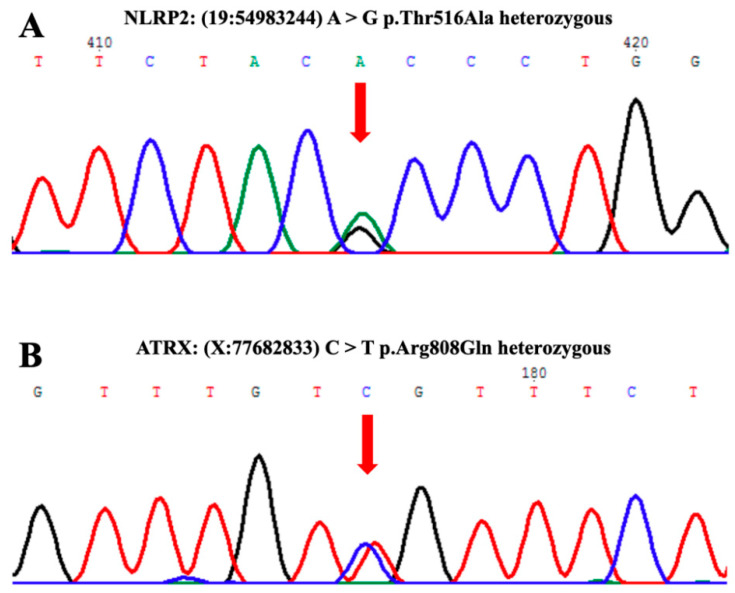
The heterozygous variants of *NLRP2* and *ATRX* identified in the patient. (**A**) Heterozygous missense mutation/variant 19:54983244 A>G (arrow), which changes threonine to alanine. (**B**) Heterozygous missense mutation/variant X:77682833 C>T (arrow), which changes arginine to glutamine.

ATRX is a chromatin remodeler in the SWI/SNF family. Previous studies have shown that it is involved in gene regulation and plays a role in maintaining the differential methylation status of imprinted genes. Additionally, mutations in *ATRX* genes are associated with an X-linked syndrome, alpha-thalassemia mental retardation syndrome (ATRX). We identified a heterozygous variant of *ATRX*, X:77682833-(C/T), in the patient. According to the gnomAD database, this variant was found in 0.3% of the 1517 samples from of the healthy Taiwanese population (Figure 6).

## 4. Discussion

There is some resemblance of the placental phenotypes of PMD and partial hydatidiform moles, but the conditions are associated with distinct clinical outcomes [5]. Differential diagnosis is necessary but can be complicated. Hydatidiform moles usually do not result in healthy babies and have an increased risk of becoming invasive moles or choriocarcinomas. The termination of pregnancy is therefore recommended. A fetus associated with PMD may be completely healthy, have growth restrictions, or have features of BWS [5,29,30]. In addition, pregnancies complicated by PMD also tend to result in fetal demise. Thus, a more precise diagnosis will help patients avoid complications and increase the opportunity to produce healthy children.

Differential diagnosis of our special case was conducted to exclude possible conditions such as PHM, twin gestation with molar pregnancy, and androgenetic/biparental mosaicism of confined placenta. Combining the results from histological examination, cytogenetic analysis, and STR analysis, we concluded that we were dealing with a PMD case. Interestingly, this reported PMD case was associated with mild to moderate trophoblast hyperplasia, which is usually characteristic of molar pregnancy (Table 2). We have confirmed that this phenotype was not caused by androgenetic-biparental mosaicism.

Up to one-third of PMD cases are associated with BWS [11]. Although the major abnormality of PMD is confined to the placenta, it has been implied that both diseases share a common pathogenic mechanism. Generally, BWS is considered an imprinting disorder involving but not limited to an imprinted locus at 11p15.5 [29,30]. This locus contains several imprinted genes, such as *CDKN1C* (*p57kip2*) and *IGF2*, and regulatory noncoding RNA *H19* and *KcNQ1OT1*, which are essential in regulating the cell cycle and in growth control. Methylation profiles of two identified imprinting control centers (ICRs) in this locus, H19-DMR (IC1) and KvDMR1 (IC2), have been extensively examined in BWS cases [31].

In BWS, the hypermethylation of H19-DMR can lead to *IGF2* loss of imprinting (LOI) and biallelic expression [32]. Furthermore, the elevation of *IGF2* results in the overgrowth of placental tissue [33,34]. Thus, in line with this evidence, *IGF2* has been suggested as a candidate gene responsible for PMD [35]. However, in our case, *H19*-DMR showed a normal differential methylation pattern in both the living baby and the placental cystic tissue. This suggests that the PMD phenotype can still arise with proper imprint marks at H19-DMR.

KvDMR1, another ICR residing in the BWS cluster, is maternally methylated. The paternal unmethylated KvDMR1 allele renders active transcription of *KCNQ1OT1* but silences *KCNQ1* and *CDKN1C* [36]. Due to parental-specific expression, a lack of *CDKN1C* expression has been used as diagnostic evidence of CHM, which does not contain the maternal genome [37]. Hypomethylation at KvDMR1 has been found to be associated with the silencing of *CDKN1C*, a cyclin-dependent kinase inhibitor, in BWS [38]. Our bisulfite sequencing analysis showed normal methylation at KvDMR1 in the whole conceptus. In addition, the immunohistochemical analysis showed positive p57 staining in the nuclei in the hydropic and enlarged villi. The data exclude the possibility of (paternal) uniparental disomy (UPD) of chromosome 11, and the deregulation of methylation imprints at KvDMR1. Taken together, in this PMD case, two ICRs residing in the BWS cluster showed proper methylation patterns and no indication of UPD, suggesting that imprinting defects of BWS were not associated with this case. This is consistent with the fact that the baby’s clinical features did not fit the criteria of BWS (e.g., macroglossia, overgrowth, or abdominal wall defect).

The baby from our PMD case had a hepatic hamartoma, which has been reported to occur in both PMD and BWS infants [60,61]. Many hepatic mesenchymal hamartoma (HMH) cases are associated with cytogenetic alterations at 19q13.4, a genomic region containing *NLRP2* and *NLRP7* [39]. *NLRP7* mutations/variants are responsible for hydatidiform mole formation and are associated with imprinting disorders [62]. As hydatidiform moles and PMD-associated HMHs are both associated with defects at 19q13, we speculated that our PMD case may also be linked to genetic alterations at that locus. Indeed, through sequencing the exons of *NLRP7* and *NLRP2* in the patient, we found an *NLRP7* mutation in exon 4 that caused a Val-to-Ile amino acid change (V319I). The *NLRP7* homozygous mutation/variant could partially account for the patient’s history of two consecutive spontaneous abortions and the PMD phenotype. Accumulated evidence indicates that these mutations and nonsynonymous variants (NSVs) of *NLRP7* are highly related to a spectrum of gestational placental diseases and spontaneous abortion [24,63]. Although NSVs of the *NLRP7* gene, including V319I, have been shown to impair IL-1 and TNF secretion, further studies are needed to establish their link to placental pathology [64]. In addition, there was a heterozygous variant of *NLRP2*, 19: 54983244-(A/G), in our patient. A germline mutation of *NLRP2* has also been linked to familial BWS [17]. Although it seems that this *NLRP2* variant did not cause BWS in the patient’s child, the hepatic hamartoma in the child, trophoblastic hyperplasia and overgrowth of the placenta may be linked with the mutation.

We also tested other imprinted loci, including *PEG10*, *PEG3*-DMR, *MEG3*-DMR, and four DMRs in the *GNAS* locus. We compared the methylation levels of DMRs between abnormal cystic placenta and adjacent normal control placenta tissues, as well as mixed normal placentae tissues from independent pregnancies with female babied of the same gestation week. We also cross reference to the published DMR patterns in human placenta [65,66,67,68,69]. Most of the examined regions had normal methylation patterns in both the defective cystic placenta regions and adjacent morphologically normal placental tissues. These results were consistent with our findings that this PMD case was not associated with a mosaicism of androgenetic/biparental tissue. In addition, NESP55-DMR at the *GNAS* locus was aberrantly hypermethylated in both cystic and adjacent normal placental tissues, whereas differential methylation was observed in the living baby, the normal control placenta, and other published reports [66]. Under normal conditions, only the paternal allele is methylated at NESP55-DMR. Since NESP55-DMR is a secondary DMR established after fertilization, the data suggest that methylation marks at AS, XLas, and 1A DMRs may be properly established in the germline, while aberrant methylation of *NESP55*-DMR occurred independently in specific cell lineages. Notably, in previous studies, complete methylation of NESP55-DMR was observed in all tested biparental hydatidiform mole (BiHM) cases [70,71,72]. NESP55 is a chromogranin-like protein expressed in neural tissue and the adrenal medulla, but its function and potential contribution to the PMD and PHM phenotypes remain to be explored [73]. Nevertheless, MEG3-DMR, another secondary DMR at the *DLK1-DIO3* imprinted locus, showed hypermethylation compared with reported results in normal human placenta [66,67,68] in the patient’s placenta. The influence of these two secondary DMRs would be part of the reason for this PMD case.

An *ATRX* heterozygous mutation, X:77682833-(C/T), was found in the patient. This point mutation was separately predicted to be tolerated or probably damaging by SIFT/PolyPhen. *ATRX* is an X-linked gene that plays roles in maintaining silencing at interstitial heterochromatic loci and imprinted genes [74]. In a mouse model, *Atrx*^null^/Y male embryos could not survive beyond 9.5 days postcoitus (dpc), resulting from the failure of extraembryonic trophoblast formation. In addition, some *Atrx*^null^*/Atrx*^WT^ females could survive but were not phenotypically normal [75]. Although we do not know the toxicity of this X:77682833-(C/T) mutation, it may be linked to this PMD case and the previous male abortion (46, XY) in this patient.

Since the living baby accompanied with PMD developed normally, untethered mesenchymal growth or focal trophoblast hyperplasia may have arisen early in trophectoderm development through aberrant genetic changes or epigenetic modifications. To this end, we identified several variants and an NSV in *NLRP7*, heterozygous variants of *NLRP2* and *ATRX*, and the hypermethylation of the secondary imprint marks NESP55 DMR and MEG3-DMR in this case. Hypermethylation or loss of expression of *MEG3* and *NESP5*5 has been observed in the products of conception of spontaneous abortions and stillbirths [76,77]. The reduced stringency of imprinting control of this PMD from both the *GNAS* and *DIK1-DIO3* loci could make the gene expression pattern in the abnormal placenta slightly closer to those in androgenetic cells. The causal relationships between *NLRP7* variants, aberrant imprints in a subset of extraembryonic lineages, and pathogenesis of PMD need to be further elucidated. *NLRP7* and *NLRP2* are reported to be maternal effect genes and are important to the regulation of imprinting. *ATRX* is also critical in maintaining imprinted gene expression during embryonic development. In this reported case, the patient had a homozygous *NLRP7* mutation/variant, an *NLRP2* heterozygous variant, and an *ATRX* heterozygous variant. These mutations may have contributed to the abnormal imprinting and placental development observed in our case. To our knowledge, this is the first discovery of the association of *NLRP7*, *NLRP2*, and *ATRX* mutations/variants and relaxed imprinting control with biparental tissue of PMD. Although it is difficult to identify the etiology of this rare human condition, increasing recognition and further examinations will improve our understanding to facilitate better diagnosis and treatments.

## Figures and Tables

**Table 1:** STR analysis of parental origin markers in the conceptus.

STR Loci	Position	Paternal	Maternal	Cystic Tissue	Living Baby	Possible Parental Origin	Rule out AG?
D3S1358	3p	15/18	15/17	15/17	15/17	Not informative	Yes
vWA	12p12-pter	17/18	16/19	17/19	17/19	Biparental	Yes
FGA	4q28	23/27	19/20	20/23	20/23	Biparental	Yes
D8S1179	8	13/13	16/17	13/17	13/17	Biparental	Yes
D21S11	21	30/31.2	30/30.2	30/30.2	30/30.2	Not informative	Yes
D18S51	18q21.3	15/17	15/17	15/17	15/17	Not informative	No
D5S818	5q21-31	10/11	11/13	11/11	11/11	Biparental	Yes
D13S317	13q22-31	8/8	11/12	8/11	8/11	Biparental	Yes
D7S820	7q	9/11	8/10	8/11	8/11	Biparental	Yes
TH01	11p15.5	7/7	6/6	6/7	6/7	Biparental	Yes
TPOX	2p23-2per	8/8	8/11	8/8	8/8	Not informative	No
CSF1PO	5q33.3-34	11/13	10/12	10/13	10/13	Biparental	Yes
D2S1338	2q35-37.1	19/19	19/24	19/19	19/19	Not informative	No
D19S433	19q12-13.1	15.2/15.2	15/15	15/15.2	15/15.2	Biparental	Yes

The STR patterns in the living baby and cystic tissue were identical in the 15 loci tested. Notably, 10 of 15 loci showed biparental origin. AG: androgenetic.

**Table 2:** The comparison of our case and other similar clinical conditions.

	PMD	PHM	Our Case
Karyotype and cellular mosaicism	Almost always diploid, often due to chimerism of androgenetic cells in the placenta [39], except for some rare cases of balanced biparental population [40], trisomy 13 [11,41] or other aneuploidy [11].	Mostly due to diandric triploidy [42,43]; some rare familial cases with diploid biparental origin [44,45,46].	46, XX Biparental, no signs of chimerism with either androgenetic or triploid cells.
Coexisting with a healthy fetus	Yes (in most cases); sometimes with congenital hemangiomas, vascular hamartomas, and hepatic mesenchymal Hamartomas [20,23,40].	Occasional. Two types: I. Twin pregnancy with one fetus and another partial mole [47,48]; II. Singleton fetus with partial mole [49,50].	Healthy baby except for a cystic lesion in the liver representing a hamartoma.
In association with IUGR, IGFD or neonatal death.	Yes [7]	Yes [51,52,53,54,55,56]	No
Distinct placental phenotype	There are aneurysmal dilated vessels on the fetal surfaces of the placentas and dilated stem villi filled with clear gelatinous material in the subchorionic region and the absence of trophoblastic hyperplasia [35].	Existence of a range of villi from normal to cystic with focal trophoblastic hyperplasia [57].	Dilated blood vessels with fibrin deposits and myxedematous with stromal fibrosis in enlarged villi and focal presence of trophoblastic hyperplasia with scalloped villous outline.
beta-hCG level	Normal or increased [1,52,58]	Greatly increased [55,59] or greatly decreased [52]	Elevated
mutations/variants for maternal effect genes	Not clear	Yes [24]	Yes
Potential altered dosages of imprinted gene expression	Yes, for all the cells of androgenetic origin and for the cases associated with BWS (15%) [13,35].	Yes, for all cases: triploidy and familial biparental diploid cases.	Relaxation of *GNAS* and *DLK1-DIO3* loci methylation.

## Data Availability

All data are available within the manuscript and upon request to the corresponding author.

## Supplementary Materials

The following are available online at https://www.mdpi.com/article/10.3390/biomedicines9050544/s1: Figure S1: Positive expression of the normally maternally expressed p57 excludes the possibility of the presence of complete androgenic tissue in the hydropic villi. Figure S2: Significant β-HCG expression in hyperplastic cytotrophoblasts. Figure S3: The presence of mesenchymal hamartoma in the living baby. Figure S4: Identification of a nonsynonymous variant in NLRP7. Table S1: Bisulfite sequencing primer list, Table S2: Genomic DNA sequencing primers.

## References

[1] Jauniaux E., Nicolaides K.H., Hustin J. (1997). Perinatal features associated with placental mesenchymal dysplasia. Placenta.

[2] Paradinas F.J., Sebire N.J., Fisher R.A., Rees H.C., Foskett M., Seckl M.J., Newlands E.S. (2001). Pseudo-partial moles: Placental stem vessel hydrops and the association with Beckwith-Wiedemann syndrome and complete moles. Histopathology.

[3] Matsui H., Iitsuka Y., Yamazawa K., Tanaka N., Mitsuhashi A., Seki K., Sekiya S. (2003). Placental mesenchymal dysplasia initially diagnosed as partial mole. Pathol. Int..

[4] Ulker V., Aslan H., Gedikbasi A., Yararbas K., Yildirim G., Yavuz E. (2013). Placental mesenchymal dysplasia: A rare clinicopathologic entity confused with molar pregnancy. J. Obstet. Gynaecol..

[5] Colpaert R.M., Ramseyer A.M., Luu T., Quick C.M., Frye L.T., Magann E.F. (2019). Diagnosis and Management of Placental Mesenchymal Disease. A Review of the Literature. Obstet. Gynecol. Surv..

[6] Moscoso G., Jauniaux E., Hustin J. (1991). Placental vascular anomaly with diffuse mesenchymal stem villous hyperplasia. A new clinico-pathological entity?. Pathol. Res. Pract..

[7] Pham T., Steele J., Stayboldt C., Chan L., Benirschke K. (2006). Placental mesenchymal dysplasia is associated with high rates of intrauterine growth restriction and fetal demise: A report of 11 new cases and a review of the literature. Am. J. Clin. Pathol..

[8] Ernst L.M. (2015). Placental Mesenchymal Dysplasia. J. Fetal Med..

[9] Himoto Y., Kido A., Minamiguchi S., Moribata Y., Okumura R., Mogami H., Nagano T., Konishi I., Togashi K. (2014). Prenatal differential diagnosis of complete hydatidiform mole with a twin live fetus and placental mesenchymal dysplasia by magnetic resonance imaging. J. Obstet. Gynaecol. Res..

[10] Van den Veyver I.B., Al-Hussaini T.K. (2006). Biparental hydatidiform moles: A maternal effect mutation affecting imprinting in the offspring. Hum. Reprod. Update.

[11] Cohen M.C., Roper E.C., Sebire N.J., Stanek J., Anumba D.O. (2005). Placental mesenchymal dysplasia associated with fetal aneuploidy. Prenat. Diagn..

[12] Kaiser-Rogers K.A., McFadden D.E., Livasy C.A., Dansereau J., Jiang R., Knops J.F., Lefebvre L., Rao K.W., Robinson W.P. (2006). Androgenetic/biparental mosaicism causes placental mesenchymal dysplasia. J. Med. Genet..

[13] Heazell A.E., Sahasrabudhe N., Grossmith A.K., Martindale E.A., Bhatia K. (2009). A case of intrauterine growth restriction in association with placental mesenchymal dysplasia with abnormal placental lymphatic development. Placenta.

[14] Duckworth R.A. (2009). Maternal effects and range expansion: A key factor in a dynamic process?. Philos. Trans. R. Soc. Lond. B Biol. Sci..

[15] Mousseau T.A., Fox C.W. (1998). The adaptive significance of maternal effects. Trends Ecol. Evol..

[16] Begemann M., Rezwan F.I., Beygo J., Docherty L.E., Kolarova J., Schroeder C., Buiting K., Chokkalingam K., Degenhardt F., Wakeling E.L. (2018). Maternal variants in NLRP and other maternal effect proteins are associated with multilocus imprinting disturbance in offspring. J. Med. Genet..

[17] Meyer E., Lim D., Pasha S., Tee L.J., Rahman F., Yates J.R., Woods C.G., Reik W., Maher E.R. (2009). Germline mutation in NLRP2 (NALP2) in a familial imprinting disorder (Beckwith-Wiedemann Syndrome). PLoS Genet..

[18] Soellner L., Begemann M., Degenhardt F., Geipel A., Eggermann T., Mangold E. (2017). Maternal heterozygous NLRP7 variant results in recurrent reproductive failure and imprinting disturbances in the offspring. Eur. J. Hum. Genet..

[19] Takahashi N., Coluccio A., Thorball C.W., Planet E., Shi H., Offner S., Turelli P., Imbeault M., Ferguson-Smith A.C., Trono D. (2019). ZNF445 is a primary regulator of genomic imprinting. Genes Dev..

[20] Kitano Y., Ruchelli E., Weiner S., Adzick N.S. (2000). Hepatic mesenchymal hamartoma associated with mesenchymal stem villous hyperplasia of the placenta. Fetal Diagn. Ther..

[21] Nelissen E.C., van Montfoort A.P., Dumoulin J.C., Evers J.L. (2011). Epigenetics and the placenta. Hum. Reprod. Update.

[22] Frost J.M., Moore G.E. (2010). The importance of imprinting in the human placenta. PLoS Genet..

[23] Tomizawa S., Sasaki H. (2012). Genomic imprinting and its relevance to congenital disease, infertility, molar pregnancy and induced pluripotent stem cell. J. Hum. Genet..

[24] Wang C.M., Dixon P.H., Decordova S., Hodges M.D., Sebire N.J., Ozalp S., Fallahian M., Sensi A., Ashrafi F., Repiska V. (2009). Identification of 13 novel NLRP7 mutations in 20 families with recurrent hydatidiform mole; missense mutations cluster in the leucine-rich region. J. Med. Genet..

[25] Edwards C.A., Ferguson-Smith A.C. (2007). Mechanisms regulating imprinted genes in clusters. Curr. Opin. Cell Biol..

[26] Tremblay K.D., Saam J.R., Ingram R.S., Tilghman S.M., Bartolomei M.S. (1995). A paternal-specific methylation imprint marks the alleles of the mouse H19 gene. Nat. Genet..

[27] Peters J., Williamson C.M. (2007). Control of imprinting at the Gnas cluster. Epigenetics.

[28] Murdoch S., Djuric U., Mazhar B., Seoud M., Khan R., Kuick R., Bagga R., Kircheisen R., Ao A., Ratti B. (2006). Mutations in NALP7 cause recurrent hydatidiform moles and reproductive wastage in humans. Nat. Genet..

[29] Weksberg R., Teshima I., Williams B.R., Greenberg C.R., Pueschel S.M., Chernos J.E., Fowlow S.B., Hoyme E., Anderson I.J., Whiteman D.A. (1993). Molecular characterization of cytogenetic alterations associated with the Beckwith-Wiedemann syndrome (BWS) phenotype refines the localization and suggests the gene for BWS is imprinted. Hum. Mol. Genet..

[30] Ping A.J., Reeve A.E., Law D.J., Young M.R., Boehnke M., Feinberg A.P. (1989). Genetic linkage of Beckwith-Wiedemann syndrome to 11p15. Am. J. Hum. Genet..

[31] Maher E.R., Reik W. (2000). Beckwith-Wiedemann syndrome: Imprinting in clusters revisited. J. Clin. Investig..

[32] Reik W., Brown K.W., Schneid H., Le Bouc Y., Bickmore W., Maher E.R. (1995). Imprinting mutations in the Beckwith-Wiedemann syndrome suggested by altered imprinting pattern in the IGF2-H19 domain. Hum. Mol. Genet..

[33] Constancia M., Hemberger M., Hughes J., Dean W., Ferguson-Smith A., Fundele R., Stewart F., Kelsey G., Fowden A., Sibley C. (2002). Placental-specific IGF-II is a major modulator of placental and fetal growth. Nature.

[34] Eggenschwiler J., Ludwig T., Fisher P., Leighton P.A., Tilghman S.M., Efstratiadis A. (1997). Mouse mutant embryos overexpressing IGF-II exhibit phenotypic features of the Beckwith-Wiedemann and Simpson-Golabi-Behmel syndromes. Genes Dev..

[35] Parveen Z., Tongson-Ignacio J.E., Fraser C.R., Killeen J.L., Thompson K.S. (2007). Placental mesenchymal dysplasia. Arch. Pathol. Lab. Med..

[36] Smilinich N.J., Day C.D., Fitzpatrick G.V., Caldwell G.M., Lossie A.C., Cooper P.R., Smallwood A.C., Joyce J.A., Schofield P.N., Reik W. (1999). A maternally methylated CpG island in KvLQT1 is associated with an antisense paternal transcript and loss of imprinting in Beckwith-Wiedemann syndrome. Proc. Natl. Acad. Sci. USA.

[37] Fisher R.A., Hodges M.D., Rees H.C., Sebire N.J., Seckl M.J., Newlands E.S., Genest D.R., Castrillon D.H. (2002). The maternally transcribed gene p57(KIP2) (CDNK1C) is abnormally expressed in both androgenetic and biparental complete hydatidiform moles. Hum. Mol. Genet..

[38] Diaz-Meyer N., Day C.D., Khatod K., Maher E.R., Cooper W., Reik W., Junien C., Graham G., Algar E., Der Kaloustian V.M. (2003). Silencing of CDKN1C (p57KIP2) is associated with hypomethylation at KvDMR1 in Beckwith-Wiedemann syndrome. J. Med. Genet..

[39] Rakheja D., Margraf L.R., Tomlinson G.E., Schneider N.R. (2004). Hepatic mesenchymal hamartoma with translocation involving chromosome band 19q13.4: A recurrent abnormality. Cancer Genet. Cytogenet..

[40] Reed R.C., Kapur R.P. (2008). Hepatic mesenchymal hamartoma: A disorder of imprinting. Pediatr. Dev. Pathol..

[41] Zhao J., Ohsumi T.K., Kung J.T., Ogawa Y., Grau D.J., Sarma K., Song J.J., Kingston R.E., Borowsky M., Lee J.T. (2010). Genome-wide identification of polycomb-associated RNAs by RIP-seq. Mol. Cell.

[42] McFadden D.E., Kalousek D.K. (1991). Two different phenotypes of fetuses with chromosomal triploidy: Correlation with parental origin of the extra haploid set. Am. J. Med. Genet..

[43] McFadden D.E., Langlois S. (2000). Parental and meiotic origin of triploidy in the embryonic and fetal periods. Clin. Genet..

[44] Vejerslev L.O., Sunde L., Hansen B.F., Larsen J.K., Christensen I.J., Larsen G. (1991). Hydatidiform mole and fetus with normal karyotype: Support of a separate entity. Obstet. Gynecol..

[45] Sunde L., Vejerslev L.O., Jensen M.P., Pedersen S., Hertz J.M., Bolund L. (1993). Genetic analysis of repeated, biparental, diploid, hydatidiform moles. Cancer Genet. Cytogenet..

[46] Deveault C., Qian J.H., Chebaro W., Ao A., Gilbert L., Mehio A., Khan R., Tan S.L., Wischmeijer A., Coullin P. (2009). NLRP7 mutations in women with diploid androgenetic and triploid moles: A proposed mechanism for mole formation. Hum. Mol. Genet..

[47] Nwosu E.C., Ferriman E., McCormack M.J., Williams J.H., Gosden C.M. (1995). Partial hydatidiform mole and hypertension associated with a live fetus--variable presentation in two cases. Hum. Reprod..

[48] Nugent C.E., Punch M.R., Barr M., LeBlanc L., Johnson M.P., Evans M.I. (1996). Persistence of partial molar placenta and severe preeclampsia after selective termination in a twin pregnancy. Obstet. Gynecol..

[49] Hsieh C.C., Hsieh T.T., Hsueh C., Kuo D.M., Lo L.M., Hung T.H. (1999). Delivery of a severely anaemic fetus after partial molar pregnancy: Clinical and ultrasonographic findings. Hum. Reprod..

[50] Dhingra K.K., Gupta P., Saroha V., Akhila L., Khurana N. (2009). Partial hydatidiform mole with a full-term infant. Indian J. Pathol. Microbiol..

[51] Jauniaux E. (1999). Partial moles: From postnatal to prenatal diagnosis. Placenta.

[52] Robertson M., Geerts L.T., de Jong G., Wainwright H. (2007). Mesenchymal dysplasia in a monochorionic diamniotic twin pregnancy with review of the differential diagnosis of cystic changes in the placenta. J. Ultrasound Med..

[53] Guven E.S., Ozturk N., Deveci S., Hizli D., Kandemir O., Dilbaz S. (2007). Partial molar pregnancy and coexisting fetus with diploid karyotype. J. Matern Fetal Neonatal Med..

[54] Sanchez-Ferrer M.L., Ferri B., Almansa M.T., Carbonel P., Lopez-Exposito I., Minguela A., Abad L., Parrilla J.J. (2009). Partial mole with a diploid fetus: Case study and literature review. Fetal Diagn. Ther..

[55] Drummond S., Fritz E. (2009). Management of a partial molar pregnancy: A case study report. J. Perinat. Neonatal Nurs..

[56] Lembet A., Zorlu C.G., Yalcin H.R., Seckin B., Ekici E. (2000). Partial hydatidiform mole with diploid karyotype in a live fetus. Int. J. Gynaecol. Obstet..

[57] McFadden D.E., Pantzar J.T. (1996). Placental pathology of triploidy. Hum. Pathol..

[58] Jalil S.S., Mahran M.A., Sule M. (2009). Placental mesenchymal dysplasia-can it be predicted prenatally? A case report. Prenat. Diagn..

[59] Berkowitz R.S., Goldstein D.P. (2009). Clinical practice. Molar pregnancy. N. Engl. J. Med..

[60] Tortoledo M., Galindo A., Ibarrola C. (2010). Placental mesenchymal dysplasia associated with hepatic and pulmonary hamartoma. Fetal Pediatr. Pathol..

[61] Cajaiba M.M., Sarita-Reyes C., Zambrano E., Reyes-Mugica M. (2007). Mesenchymal hamartoma of the liver associated with features of Beckwith-Wiedemann syndrome and high serum alpha-fetoprotein levels. Pediatr. Dev. Pathol..

[62] Slim R., Wallace E.P. (2013). NLRP7 and the Genetics of Hydatidiform Moles: Recent Advances and New Challenges. Front. Immunol..

[63] Messaed C., Chebaro W., Di Roberto R.B., Rittore C., Cheung A., Arseneau J., Schneider A., Chen M.F., Bernishke K., Surti U. (2011). NLRP7 in the spectrum of reproductive wastage: Rare non-synonymous variants confer genetic susceptibility to recurrent reproductive wastage. J. Med. Genet..

[64] Messaed C., Akoury E., Djuric U., Zeng J., Saleh M., Gilbert L., Seoud M., Qureshi S., Slim R. (2011). NLRP7, a nucleotide oligomerization domain-like receptor protein, is required for normal cytokine secretion and co-localizes with Golgi and the microtubule-organizing center. J. Biol. Chem..

[65] Hamada H., Okae H., Toh H., Chiba H., Hiura H., Shirane K., Sato T., Suyama M., Yaegashi N., Sasaki H. (2016). Allele-Specific Methylome and Transcriptome Analysis Reveals Widespread Imprinting in the Human Placenta. Am. J. Hum. Genet..

[66] Hanna C.W., Penaherrera M.S., Saadeh H., Andrews S., McFadden D.E., Kelsey G., Robinson W.P. (2016). Pervasive polymorphic imprinted methylation in the human placenta. Genome Res..

[67] Hiura H., Hattori H., Kobayashi N., Okae H., Chiba H., Miyauchi N., Kitamura A., Kikuchi H., Yoshida H., Arima T. (2017). Genome-wide microRNA expression profiling in placentae from frozen-thawed blastocyst transfer. Clin. Epigenetics.

[68] Nelissen E.C.M., Dumoulin J.C.M., Daunay A., Evers J.L.H., Tost J., van Montfoort A.P.A. (2013). Placentas from pregnancies conceived by IVF/ICSI have a reduced DNA methylation level at the H19 and MEST differentially methylated regions. Hum. Reprod..

[69] Vasconcelos S., Ramalho C., Marques C.J., Doria S. (2019). Altered expression of epigenetic regulators and imprinted genes in human placenta and fetal tissues from second trimester spontaneous pregnancy losses. Epigenetics.

[70] Judson H., Hayward B.E., Sheridan E., Bonthron D.T. (2002). A global disorder of imprinting in the human female germ line. Nature.

[71] El-Maarri O., Seoud M., Coullin P., Herbiniaux U., Oldenburg J., Rouleau G., Slim R. (2003). Maternal alleles acquiring paternal methylation patterns in biparental complete hydatidiform moles. Hum. Mol. Genet..

[72] Kou Y.C., Shao L., Peng H.H., Rosetta R., del Gaudio D., Wagner A.F., Al-Hussaini T.K., Van den Veyver I.B. (2008). A recurrent intragenic genomic duplication, other novel mutations in NLRP7 and imprinting defects in recurrent biparental hydatidiform moles. Mol. Hum. Reprod..

[73] Plagge A., Isles A.R., Gordon E., Humby T., Dean W., Gritsch S., Fischer-Colbrie R., Wilkinson L.S., Kelsey G. (2005). Imprinted Nesp55 influences behavioral reactivity to novel environments. Mol. Cell Biol..

[74] Voon H.P., Hughes J.R., Rode C., De La Rosa-Velazquez I.A., Jenuwein T., Feil R., Higgs D.R., Gibbons R.J. (2015). ATRX Plays a Key Role in Maintaining Silencing at Interstitial Heterochromatic Loci and Imprinted Genes. Cell Rep..

[75] Garrick D., Sharpe J.A., Arkell R., Dobbie L., Smith A.J., Wood W.G., Higgs D.R., Gibbons R.J. (2006). Loss of Atrx affects trophoblast development and the pattern of X-inactivation in extraembryonic tissues. PLoS Genet..

[76] Lin S.P., Coan P., da Rocha S.T., Seitz H., Cavaille J., Teng P.W., Takada S., Ferguson-Smith A.C. (2007). Differential regulation of imprinting in the murine embryo and placenta by the Dlk1-Dio3 imprinting control region. Development.

[77] Pliushch G., Schneider E., Weise D., El Hajj N., Tresch A., Seidmann L., Coerdt W., Muller A.M., Zechner U., Haaf T. (2010). Extreme methylation values of imprinted genes in human abortions and stillbirths. Am. J. Pathol..

