# Supplementary material for: Variants in Maternal Effect Genes and Relaxed Imprinting Control in a Special Placental Mesenchymal Dysplasia Case with Mild Trophoblast Hyperplasia
Source: Biomedicines. 2021 May 13;9(5):544. doi: 10.3390/biomedicines9050544 (PMC8152467; doi:10.3390/biomedicines9050544)
Supplement: Supplementary file 1 [file biomedicines-09-00544-s001.zip › biomedicines-1151845-SI.pdf]

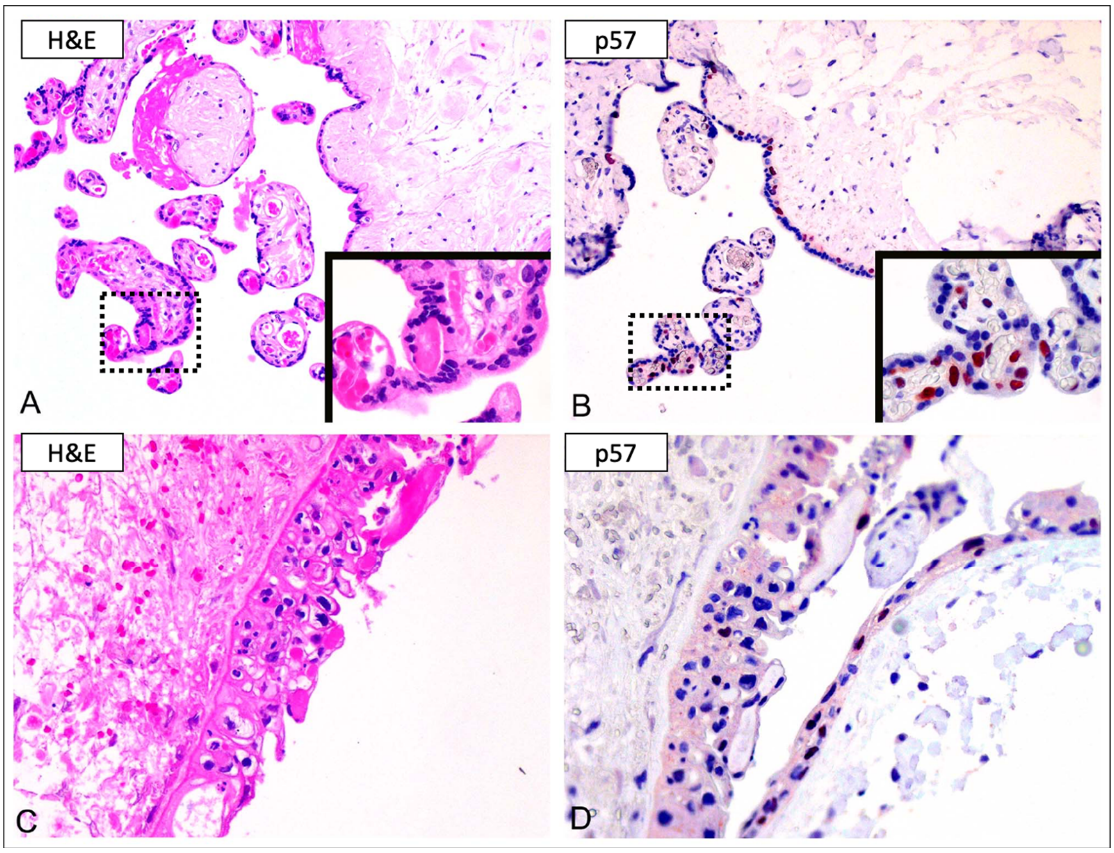

Figure S1. Positive expression of the normally maternally expressed p57 excludes the possibility of the presence of complete androgenic tissue in the hydropic villi. (A-B) Both hydropic villi (100X and inset, 400X). C-D) Enlarged villi were positive for p57 (cyclin-dependent kinase inhibitor 1C) staining in the nuclei (200X).

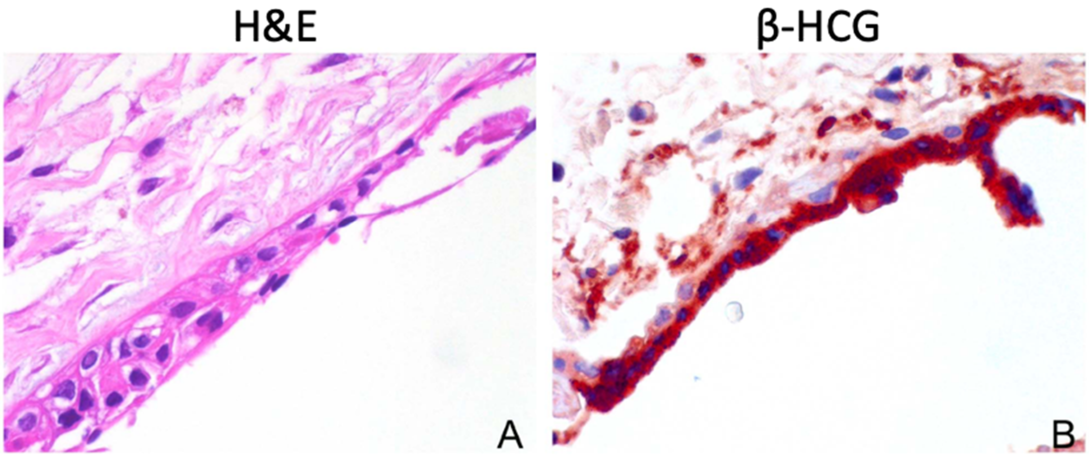

Figure S2. Significant β-HCG expression in hyperplastic cytotrophoblasts.

(A) H&E staining of hyperplastic trophoblasts (most are cytotrophoblasts, 400X). (B)

Hyperplastic trophoblasts are positive for β-HCG (human chorionic gonadotropin β-subunit) (hematoxylin stain, 400X). Microscopic (histological) descriptions: Cytotrophoblasts: small, round mononuclear cells with distinct cell borders, minimal clear or eosinophilic cytoplasm and single vesicular nuclei; Syncytial trophoblasts: multinucleated giant cells with abundant eosinophilic or basophilic cytoplasm, often with multiple intracytoplasmic vacuoles and dense pyknotic nuclei.

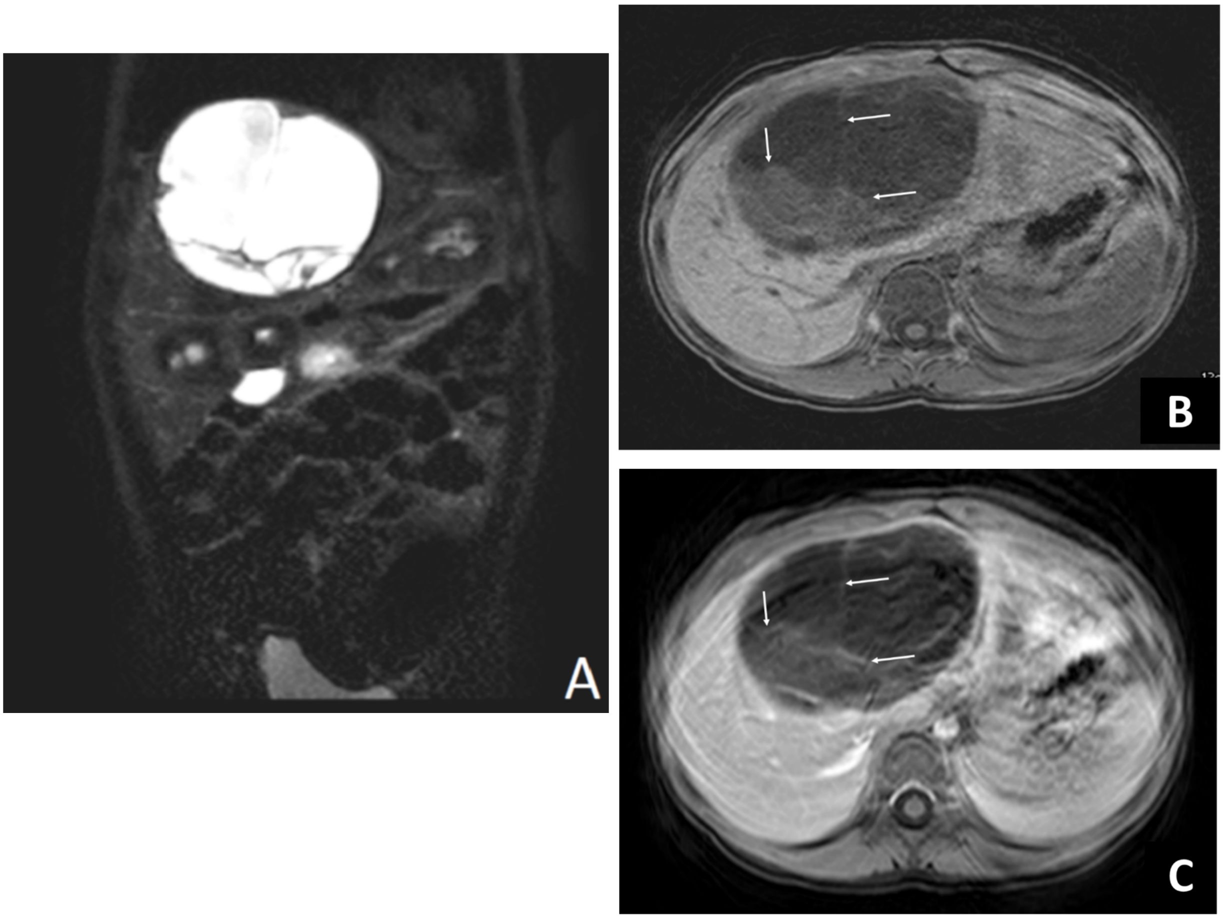

Figure S3. The presence of mesenchymal hamartoma in the living baby.

(A) Abdominal MRI coronal T2WI with fat suppression revealed multiple hepatic cystic nodules and masses compatible with the image pattern of a mesenchymal hamartoma. (B-C) Abdominal MRI axial images, including T1WI and contrast-enhanced T1WI at the level of a large hepatic mass, showed most cystic components and some enhancing inner septae (white arrows).

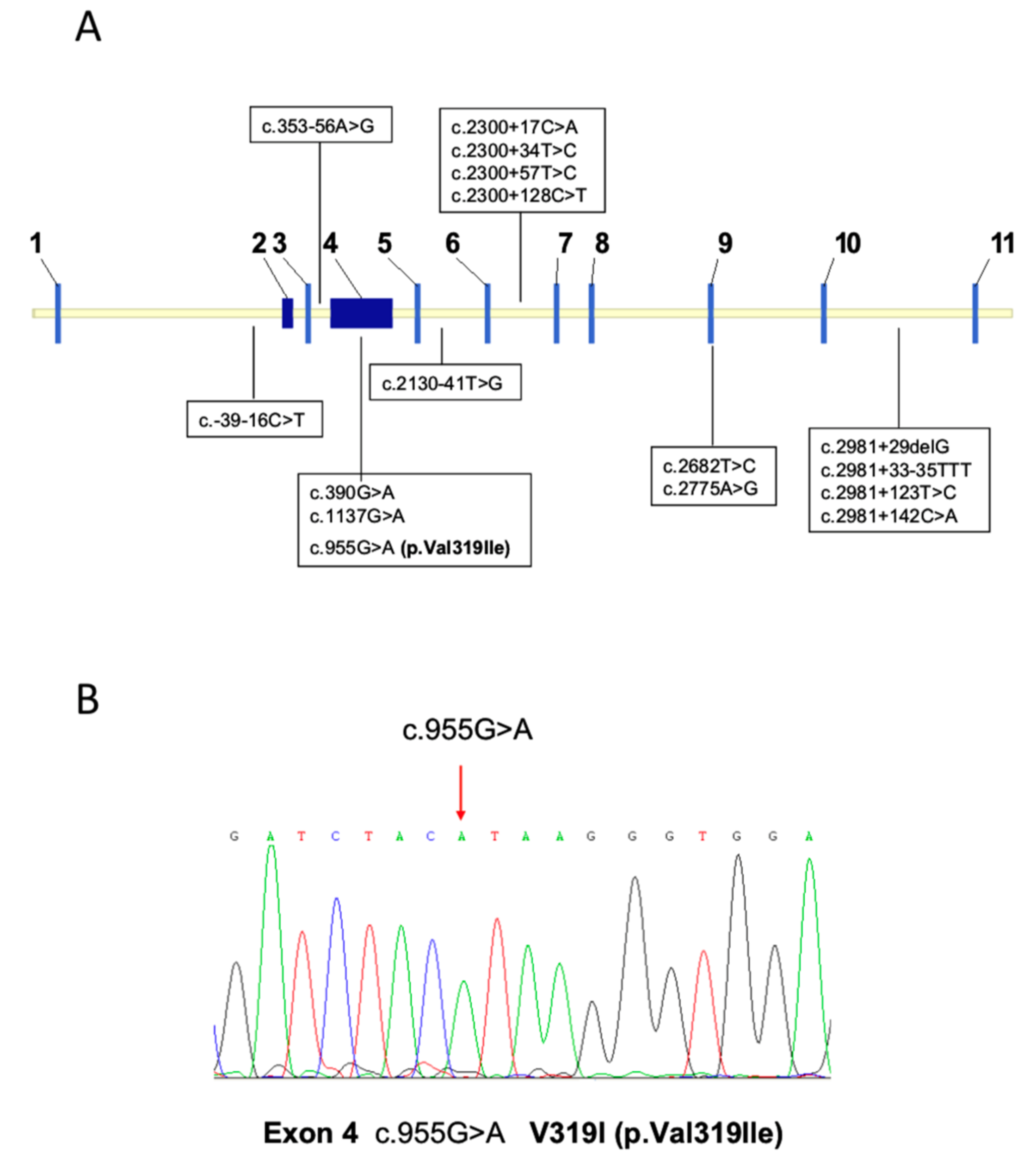

Figure S4. Identification of a nonsynonymous variant in NLRP7.

(A) Schematic representation of NLRP7 (NACHT, LRR and PYD domain-containing protein 7) mutations/variants identified in the patient compared to NC_000019.8. **(B)** Missense mutation c.955G>A in exon 4, resulting in an amino acid change from valine to isoleucine.

Supplemental Table I

Bisulfite sequencing primer list

Primer name	Accession number	Primer sequence (5´→3´)	Band size (bp)	Tm	Reference
KvDMR	AJ006345	F-TGATGTGTTTATTATTTYGGGG<br>R-CCCTAAAATCCCAAATCCTC	304	55	[1]
ZIM2/PEG3	AC006115	F-AAAAGGTATTAATTATTTATAGTTTGGT<br>R-AAAAATATCCACCCTAAACTAATAA	322	53	[2]
SNRPN	AC009696	F-GGTTTTTTTTTATTGTAATAGTGTTGTGGGG<br>R-CTCCAAAACAAAAAACTTTAAAACCCAAATTC	409	51	[2]
SNRPN	AC009696	F-AGGTTTTTTTTTATTGTAATAGTGTTGTGGGG<br>R-TCCAAAACAAAAAACTTTAAAACCCAAATTC	355	67	[3]
PEG10	AC069292	F- GTGTTATGTTTTATAAATAGATAAG<br>R- AACTCATATACCTCTACAATTC	375	53	[4]
H19	AF087017	F-TGTATAGTATATGGGTATTTTTGGAGGTTT<br>R-TCCTATAAATATCCTATTCCCAAATAACC	231	54	[2]
GNAS1-1A	AL121917	F-TTTTGTTTTTTTTTYGTTTGTTTAT<br>R-ACAACTTCRACAACCACCTCRACAAC	219	55	[2]
GNAS1-AS	AL121917	F- AATTTGTGGTATGAGGAAGAGTGAT<br>R- TCAACCATTAAACAAAAATCATACC	235	58	Self-design
GNAS1-XLαs	AL121917	F-AGGTTTGTAAAGGTTGGTTTTAGAG<br>R- AAATCTCCATCCAAAAACTACTACC	298	51	Self-design
NESP55	AL12191	F-TTTTTGTAGAGTTAGAGGGTAGGT	343	55	[2]

	7	R-AAATAAAACAACTCAAAATCTACC			
IGDMR (CG4)	NT_0264 37	F- TTTTATTATTGAATTGGGTTTGTTAGT<br>R- ACAATTCCTACTACAAAATTTCAACA	311	57	[5]
GTL2-DMR (CG7)	NT_0264 37	F- TTGTGTTTGAATTTATTTTGTTT<br>R- CCCCAAATTCTATAACAAATTACT	168	55	[5]

PCR program:

95: 5 min--(95: 30 sec--X: 45 sec--72: 1 min) ×40--72: 10 min—4

Supplemental Table II Genomic DNA sequencing primers

Primer name	Region	Primer sequence (5´→3´)	Band size (bp)
NLRP2	Exon1	CACTGCTAACTTATTTTTCAT<br>TGGCCTTCTGAATTTCTAAA	556
	Exon2	GGAGATAAGAGAAAGGAACAA<br>CCAGATCTCACATGAAGAATA	208
	Exon3	CTAATCTAGGCTTCACTACT<br>TTCTTCCCTCTATTCTTACG	352
	Exon4	CACATTTGTAGCTTATTTGC<br>ATAAATGCTTTGTGTTACATAG	305
	Exon5A	CTCTCCCTTCCCTCCTCACC<br>GCTTGGGCTAGGATGTGTGG	406
	Exon5B	AGCTGGTCTTCAGGGACTGG<br>GCAGAGGAAACGCAGGAACA	524
	Exon5C	CTGCAGATGGAGAAGGGGGA<br>AGCGAGGCCAAAGGAGTAGT	456
	Exon5D	ACCCTGGAGAAGGAGGAGGA<br>TAAGGGGGCATGGGATGAGG	544
	Exon6	CAGCCGTGATGGACACACAT<br>GGACAGGACTCTCTCAATTCCC	357
	Exon7	CGAGCCCCTGGTTTCCATTT<br>CTGCATCCTGGGTCATCTGC	378
Primer	Region	Primer sequence (5´→3´)	Band size

name			(bp)
NLRP2	Exon8	ACGTGGTCCTATTTCTCCCACA<br>TCAGGCATGACCATTGCTCG	267
	Exon9	GCTAGCCGGGAAGGTTGAAG<br>ACCCTACTCAAACCCGGAGG	306
	Exon10	ACTCACAGGTTCGGGTTTGC<br>GGGAAGTCGGCTTCACTGAT	302
	Exon11	GCACTGGCTGCATTAACGTG<br>CAGTCTCCTACAGCAGGTCCA	300
	Exon12	CAGGAAAGGTGACCCATGCC<br>TCTTTTCTCTAACTTGTCAAAGCCCA	538

PCR program: 95: 5 min--(95: 30 sec--55: 45 sec--72: 1 min) ×35--72: 10 min—4

1. Monk, D.; Arnaud, P.; Apostolidou, S.; Hills, F.A.; Kelsey, G.; Stanier, P.; Feil, R.; Moore, G.E. Limited evolutionary conservation of imprinting in the human placenta. *Proc Natl Acad Sci U S A* **2006**, *103*, 6623-6628, doi:10.1073/pnas.0511031103.
2. Judson, H.; Hayward, B.E.; Sheridan, E.; Bonthron, D.T. A global disorder of imprinting in the human female germ line. *Nature* **2002**, *416*, 539-542, doi:10.1038/416539a.
3. El-Maarri, O.; Seoud, M.; Coullin, P.; Herbiniaux, U.; Oldenburg, J.; Rouleau, G.; Slim, R. Maternal alleles acquiring paternal methylation patterns in biparental complete hydatidiform moles. *Hum Mol Genet* **2003**, *12*, 1405-1413, doi:10.1093/hmg/ddg152.
4. Monk, D.; Wagschal, A.; Arnaud, P.; Muller, P.S.; Parker-Katiraee, L.; Bourc'his, D.; Scherer, S.W.; Feil, R.; Stanier, P.; Moore, G.E. Comparative analysis of human chromosome 7q21 and mouse proximal chromosome 6 reveals a placental-specific imprinted gene, TFPI2/Tfpi2, which requires EHMT2 and EED for allelic-silencing. *Genome Res* **2008**, *18*, 1270-1281, doi:10.1101/gr.077115.108.

5. Kagami, M.; Sekita, Y.; Nishimura, G.; Irie, M.; Kato, F.; Okada, M.; Yamamori, S.; Kishimoto, H.; Nakayama, M.; Tanaka, Y.; et al. Deletions and epimutations affecting the human 14q32.2 imprinted region in individuals with paternal and maternal upd(14)-like phenotypes. *Nat Genet* **2008**, *40*, 237-242, doi:ng.2007.56 [pii]
10.1038/ng.2007.56.
